# Whole-genome resequencing and transcriptomic analysis of genes regulating anthocyanin biosynthesis in black rice plants

**DOI:** 10.1007/s13205-018-1140-3

**Published:** 2018-02-07

**Authors:** Jae-Hyeon Oh, Ye-Ji Lee, Eun-Ju Byeon, Byeong-Chul Kang, Dong-Soo Kyeoung, Chang-Kug Kim

**Affiliations:** 1 Genomics Division, National Institute of Agricultural Sciences, Jeonju, 54874 Korea; 2 Department of Environmental Resources, Sangmyung University, Cheonan, 31066 Korea; 3 Department of Crop Science and Biotechnology, Chonbuk National University, Jeonju, 54896 Korea; 4 Codes Division, Insilicogen Inc., Suwon, 16954 Gyeonggi-do Korea

**Keywords:** Anthocyanin biosynthesis, Black rice, Rice resequencing, Transcriptomic analysis

## Abstract

Anthocyanins are involved in many diverse functions in rice, but their benefits have yet to be clearly demonstrated. Our objective in this study was to identify anthocyanin-related genes in black rice plants. We identified anthocyanin-related genes in black rice plants using a combination of whole-genome resequencing, RNA-sequencing (RNA-seq), microarray experiments, and reverse-transcriptase polymerase chain reaction (RT-PCR). Using multi-layer screening from 30 rice accessions, we identified 172,922 single-nucleotide polymorphisms (SNPs) and 1276 differentially expressed genes that appear to be related to anthocyanin biosynthesis. We identified 18 putative genes from 172,922 SNPs using intensive selective sweeps. The 18 candidate genes identified from SNPs were not significantly correlated with the RNA-seq expression pattern or other well-known anthocyanin biosynthesis/metabolism genes. We also identified nine putative genes from 1276 differentially expressed genes using RNA-seq transcriptome analysis. In addition, we identified four phylogenetic groups from these nine candidate genes and 51 pathway-network genes. Finally, we verified nine anthocyanin-related genes using a newly designed microarray and semi-quantitative RT-PCR. We suggest that these nine identified genes appear to be related to the regulation of anthocyanin biosynthesis and/or metabolism.

**Electronic supplementary material:**

The online version of this article (10.1007/s13205-018-1140-3) contains supplementary material, which is available to authorized users.

## Introduction

Anthocyanins are involved in many diverse functions, but their benefits have yet to be clearly demonstrated (Mateus and de Freitas 2008; Shi and Xie 2014; Fernandes et al. 2015). In rice (*Oryza sativa* L.), anthocyanins are found in the aleurone layer of black rice and in the leaves of colored rice. Black rice pigments contain high levels of anthocyanins (Kim et al. 2011), which accumulate to give the grain its black color (He and Giusti 2010). Colored rice produces an anthocyanin that is associated with the red and purple coloration of the leaves (Kim et al. 2008). Anthocyanins are often used to indicate the health index of foods due to their antioxidant properties, which play important roles in preventing cancer, inflammation, and cardiovascular disease; controlling obesity; and alleviating diabetes (Kim et al. 2011; He and Giusti 2010; Kim et al. 2008; Kong et al. 2003).

In previous studies, anthocyanin-related genes have been identified as important regulators that utilize the middle steps of the flavonoid-biosynthetic pathway (Shih et al. 2008; Du et al. 2009; Shao et al. 2011). As next-generation sequencing technology advances, genome resequencing can reveal genomic variations, evolutionary history, and population structure, and can identify genomic loci responsible for phenotypic and physiological traits (Xu et al. 2012). Similarly, RNA sequencing (RNA-seq) and microarray analysis have been used to determine gene expression for genome-wide transcriptome profiling (Zhao et al. 2014; Mantione et al. 2014). Analysis of gene regulation related to anthocyanin biosynthesis has identified various gene families and transcriptome genes (Oikawa et al. 2015; Sweeney et al. 2006; Furukawa et al. 2007). The plant portal Gramene (http://www.gramene.org/) reports that the rice genome contains 15 genes involved in anthocyanin biosynthesis, and the Kyoto Encyclopedia of Genes and Genomes (KEGG, http://www.genome.jp/kegg/) database reports 14 orthologous gene groups within the anthocyanin pathway.

Here, we report the identification of anthocyanin-related genes in black rice plants using genome resequencing, RNA-seq, and microarray experiments with reverse-transcriptase polymerase chain reaction (RT-PCR) verification.

## Methods

### Rice materials and experimental design

**Fig. 1:**
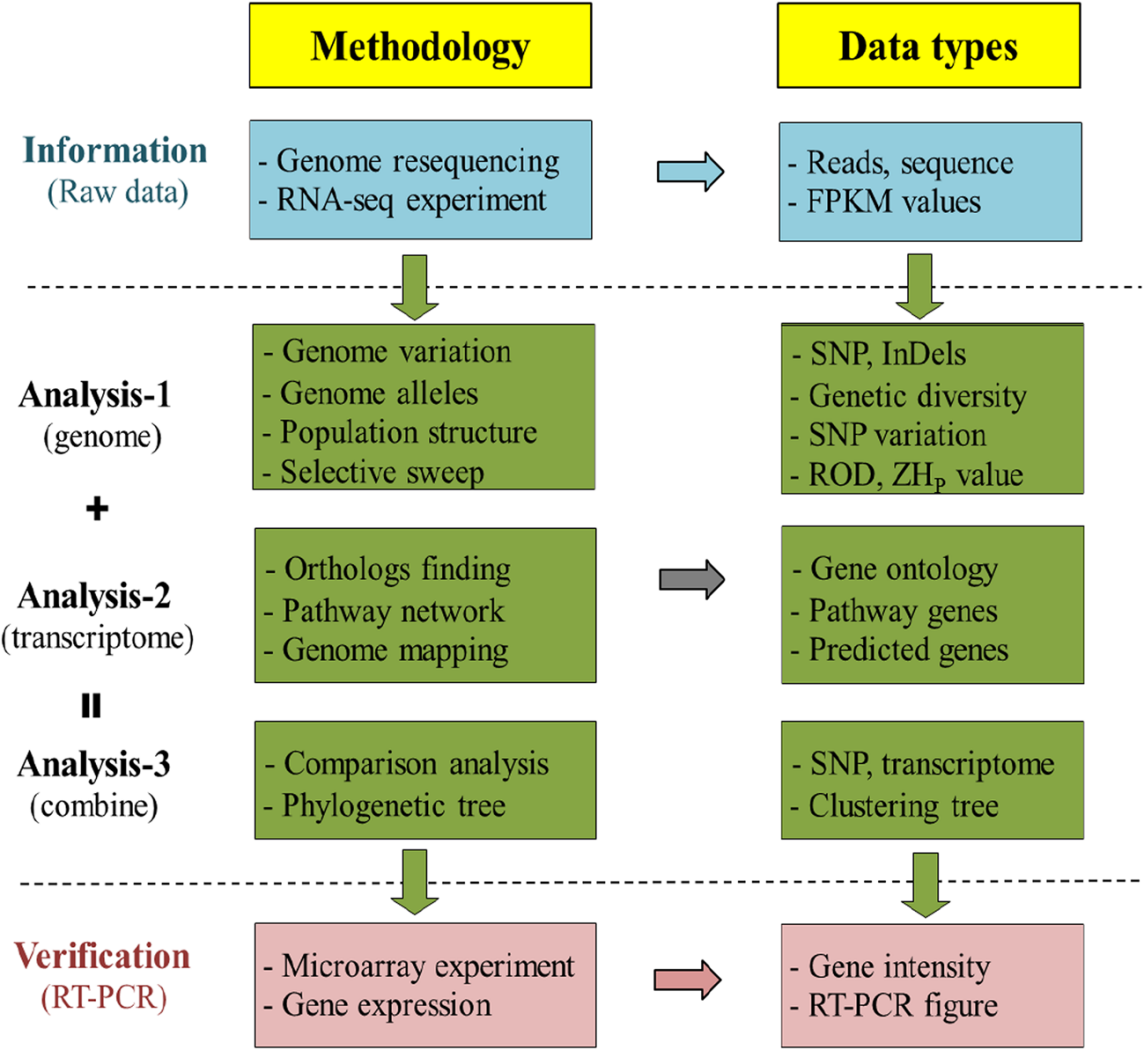
Flowchart of our screening strategy. *RNA-seq* RNA-sequencing, *FPKM* fragments per kilobase of transcript per million mapped reads, *SNP* single-nucleotide polymorphism, *ROD* reduction of diversity, *ZHp* Z-transformed heterozygosity, *RT-PCR* reverse-transcriptase polymerase chain reaction

We conducted a three-step investigation (i.e., information, analysis, and verification) to identify rice genes involved in anthocyanin biosynthesis (Fig. 1). In the first step, we conducted resequencing on 17 rice accessions (eight black rice and nine white rice accessions). In the second step, we conducted transcriptome analysis on 10 accessions (eight black rice and two white rice accessions) selected from the 17 accessions in step 1. In the third step, we performed a microarray experiment with three accessions (two black rice accessions and one white rice accession) selected from the 10 accessions in step 2 (Table 1).

**Table 1:** Rice accessions used in this study for resequencing, RNA-sequencing, and microarray experiments

Type	Group	Name	Accession ID^a^	Taxonomy	Phenotype^b^
Re-sequencing	High	A1 (AE1)	ERP009995	*O. Sativa* var. *japonica*	Seed
		A2 (BE1)	ERP009998		Seed
		A3 (AM2)	ERP009996		Seed
		A4 (BM2)	ERP009999		Seed
		A5 (AL3)	ERP009997		Seed
		A6 (BL3)	ERP010000		Seed
		A7 (Jado)	ERP008700		Leaves
		A8 (D101)	ERP008715		Leaves
	None	N1 (CA1)	ERP010001	*Japonica*	Seed, leaves
		N2 (CB2)	ERP010002		Seed, leaves
		N3 (DJ1)	ERP010001		Seed, leaves
		N4 (KH)	ERP049282		Seed, leaves
		N5 (DJ2)	ERP008697		Seed, leaves
		N6 (HY)	ERP001620		Seed, leaves
		N7 (BLB)	ERP001655		Seed, leaves
		N8 (HY-04)	ERP001653		Seed, leaves
		N9 (HY-08)	ERP001654		Seed, leaves
RNA-seq	High	AR1-AR6^c^	ERP009858-9904	*Japonica*	Seed
		AR7	ERP008777-8779		Leaves
		AR8	ERP008789,8808,8791		Leaves
	None	NR3	ERP009898-9900	*Japonica*	Seed, leaves
		NR5	ERP008763-8765		Seed, leaves
Microarray	High	AM3^d^	IT218587^e^	*Japonica*	Seed, leaves
		AM7	IT210918		
	None	NM3	IT235273	*Japonica*	Seed, leaves

^a^Registered sample name and accession number of EMBL-EBI (http://www.ebi.ac.uk)

^b^Phenotype indicates the organ for anthocyanin accumulation

^c^Number and first character indicate the same accession of resequencing accessions. For example, AR6 is the RNA-seq data of the A6 (BL3) accession, and NR3 is the RNA-seq of the N3 (DJ1) accession

^d^As in the case of RNA-seq, AM3 is the microarray data of the A3 (AM2) accession

^e^Accession number from the RDA-Genebank, Korea (http://www.genebank.go.kr/)

We collected resequencing information for 17 rice accessions from EMBL-EBI (http://www.ebi.ac.uk) and NCBI-Genbank (https://www.ncbi.nlm.nih.gov/genbank/). We characterized the 17 accessions as either high-anthocyanin accessions (eight accessions) or non-anthocyanin accessions (nine accessions) based on the leaf- and seed-colored rice accessions. We performed RNA-seq using 10 selected accessions (eight high-anthocyanin and two non-anthocyanin accessions) from the 17 rice accessions described above. The non-replicated transcriptome was sequenced for each of the 10 accessions at three time points (i.e., at 5, 10, and 15 days after the heading stage). These time intervals were chosen because tissue differentiation events occur early in the pericarp during rice seed development (Wu et al. 2016). Using a newly designed microarray, we conducted a total of 27 microarray expression experiments on three accessions assayed in triplicate at the same three time points as those used in the RNA-seq experiments. The characteristics of these accessions are reported in Table 1.

### Preprocessing and detection of SNPs

Pre-processed reads were aligned to a rice reference, which was necessary for running the Genome Analysis Toolkit (GATK, https://software.broadinstitute.org/gatk/) using the Bowtie2 (http://bowtie-bio.sourceforge.net/bowtie2/) program. For variant calling, duplicate reads were removed and alignment files were coordinate-sorted via Picard (v1.105, http://picard.sourceforge.net/). We then called variants individually on each sample using the HaplotypeCaller/GATK. To reduce erroneous SNPs, we applied the Hardy–Weinberg equilibrium (HWE), which tests genetic variation within a population (Sidore et al. 2015), and filtered out 280 low-quality mapping regions that failed the HWE test (*p* > 0.001) with a minor allele frequency (MAF) > 0.1 (McNally et al. 2009; Sidore et al. 2015). To provide insight into the molecular evolution of the selected SNPs, we identified transitions (changes from A <–> G and C <–> T), 282 transversions (changes from A <–> C, A <–> T, G <–> C, or G <–> T), and also the ratio of the transitions to transversions for 283 pairs of sequences.

### Determination of population structure based on SNPs

We analyzed the population structure using FRAPPE software (http://med.stanford.edu/tanglab/software/) based on the maximum-likelihood method. The population structure was performed on the rice accessions using the SNPs that passed the HWE test. We divided individual accessions into *K* clusters based on a maximum-likelihood method (Chen et al. 2014). The genotype information of all samples was converted to a PED file, and Principal Component Analysis (PCA) was performed in R.

### Gene detection based on selective sweeps

In genetics, a selective sweep occurs when a beneficial allele increases in frequency rapidly due to strong natural selection, leading to less variation among nearby linked alleles. To detect the genomic areas in which selective sweeps had occurred due to artificial selection, we calculated reduction of diversity (ROD) scores based on the ratio of diversity between the high-anthocyanin and non-anthocyanin accessions (Xu et al. 2012). We calculated the ROD scores based on *π*_high_ (the *π* value of the high-anthocyanin accessions) and *π*_none_ (the *π* value of the non-anthocyanin accessions) using the following equation:

1 $$ {\text{ROD}} = 1 - \left( {{{\pi_{\text{high}} } \mathord{\left/ {\vphantom {{\pi_{\text{high}} } {\pi_{\text{none}} }}} \right. \kern-0pt} {\pi_{\text{none}} }}} \right) $$

where the population parameter *π* is the average number of nucleotide differences between any two DNA sequences. We divided the entire genome into 10, 50, 100, and 500-kb windows and calculated the ROD score for each window. We screened the candidate genes in selective-sweep regions based on the significance level (*p* ≤ 0.01) of the ROD distribution. In addition, we compared the high-anthocyanin accessions (*π*_high_), non-anthocyanin accessions (*π*_none_, control), and the ROD between the two groups using the Circos program (Circos, Vancouver, BC, Canada). To visualize selective sweeps, we generated Manhattan plots using the allele counts of identified SNP positions in 100-kb sliding windows along the genome with a step size of 20 kb. To detect putatively selected regions, we applied a threshold of Z-transformed heterozygosity (ZHp)  ≤  − 1.5. We calculated the ZHp score using two variables, such as the frequency of the most common allele and the frequency of the least common allele (Kong et al. 2003).

### Gene expression analysis

For the RNA-seq analysis of 10 rice accessions, we performed quality control on the raw sequence data using FastQC. Useful transcripts were predicted using CLC Assembly Cell 3.2 (CLC Bio, Aarhus, Denmark) and the Trinity software package (http://trinityrnaseq.sourceforge.net/). We calculated the fragment per kilobase of transcript per million mapped reads (FPKM) score for the transcribed fragments. To compare gene expression levels among the rice accessions, we screened the candidate genes using the FPKM scores based on twofold or greater increases or decreases in FPKM values between the high-anthocyanin and non-anthocyanin accessions at the three time points described above. We conducted a GO-enrichment analysis using GoMiner (National Cancer Institute, http://discover.nci.nih.gov/gominer/). BLASTP analysis was performed to find a tentative counterpart to the rice gene in the *Arabidopsis* genome. False discovery rate (FDR) values were obtained from 100 randomizations. GO terms for which the FDR was < 0.05 in at least one group were collected. We categorized each gene using the expression intensity and GO function, and identified false discoveries using one-sided Fisher’s exact tests (*p* value ≤ 0.05).

### Microarray experiments

To verify the expression patterns of the anthocyanin-related genes, we designed a microarray based on the genome information of IRGSP_1_0 (http://rapdb.lab.nig.ac.jp). The alternatively spliced transcript detection microarray covered 36,176 loci and 40,139 transcripts. Using this newly designed microarray, we performed a total of 27 experiments from three rice accessions assayed in triplicate at three time points (i.e., 5, 10, and 15 days after the heading stage). We scanned the microarray for Cy3 signals with the Genepix 4000B Scanner (Axon Instruments, CA, USA), and digitized the signals using Nimblescan (Roche NimbleGen, Inc., USA). To compare the expression levels among the rice accessions, we screened the anthocyanin-related genes for significant (at least twofold) changes in expression levels between high-anthocyanin and non-anthocyanin accessions at the three time points.

### Genome mapping of the pathway-network genes

To identify pathway networks of interacting genes, we first used MedScan Reader (Ariadne Inc., Rockville, MD, USA) to extract genes from the anthocyanin biosynthesis pathway. For enriched-pathway analysis, we determined the most significant network interactions with a Fisher’s exact test (*p* value ≤ 0.05) using the Pathway Studio^®^ software (Ariadne Inc., Rockville, MD, USA). To predict the functions of the selected genes, we identified the most likely chromosomal positions of each gene using the FSTVAL program (GGBio Inc., Yongin, Korea). We found the best mapping position using the BLASTN tool (https://blast.ncbi.nlm.nih.gov/) with an *e* value cutoff of ≤ 1.0 × 10^−5^. We anchored each gene position by a mapping calculation to select the highest scoring region within the rice genome.

### Phylogenetic analysis for hierarchical clustering

For the phylogenetic analysis, we aligned the amino acid sequences of the candidate genes involved in anthocyanin-related pathways with the ClustalW method using the slow-accurate options in DNASTAR Lasergene^®^ v8.1 (DNASTAR, Inc., Madison, WI, USA), We trimmed the aligned sequences at both ends to eliminate regions of poor alignment (Adelskov and Patel 2016). We then constructed phylogenetic trees using the maximum-likelihood algorithm implemented within the MEGA6 software (http://www.megasoftware.net/). We tested the phylogeny of each node by bootstrapping with 1000 replicates.

### RNA extraction and semi-quantitative RT-PCR

We extracted the total RNA from rice plants using the RNeasy plant kit from Qiagen (Qiagen, Inc., Hilden, Germany). We verified the concentration and quality of the RNA samples using a NanoDrop^®^ ND-1000 Spectrophotometer (NanoDrop Technologies, Wilmington, DE, USA). All PCR primers were designed using the Primer3 software (https://sourceforge.net/projects/primer3/). The PCR amplification conditions were: initial denaturing for 5 min at 94 °C; followed by 35 cycles of denaturation (1 min at 95 °C), annealing (30 s at 60 °C), and extension (1 min 30 s at 72 °C); and a final extension for 7 min at 72 °C. The DNA of downregulated genes was also evaluated to verify whether primers were not working due to sequence mismatches. DNA extraction and experiments were performed using the same method as the RNA method.

## Results and discussion

### Genome resequencing for SNP detection

To identify anthocyanin-related genes, we collected 17 rice accessions, which included nine accessions that do not produce anthocyanins (non-anthocyanin accessions) and eight accessions that produce high levels of anthocyanins (high-anthocyanin accessions). We mapped the preprocessed reads of these 17 accessions to the rice IRGSP-1.0 reference genome (http://rapdb.dna.affrc.go.jp/). The total read count was 2.74 billion reads (254.9 Gbp of nucleotides). On average, we generated 160.9 million reads per accession. The guanine–cytosine (GC) content ranged from 37.9 to 44.6%. The mapping percentage per accession was 97.5%. Therefore, we assumed that the resequencing data were sufficient for subsequent analysis.

To investigate genome variation, we first identified a total of 1176,226 unique SNPs using the resequencing reads of the 17 accessions. Then, we screened 653,065 bi-allelic SNPs, in which exactly two alleles were observed. Finally, we identified a total of 172,922 SNPs after applying the HWE test.

The number of variants within each chromosome ranged from 5783 to 34,371. Chromosome 2 contained the most variants and chromosome 12 contained the fewest variants. Most of the SNPs were located in intergenic (34.9%), upstream (25.1%), or downstream (22.9%) regions rather than in exons or introns. To predict the impact of amino acid changes on the 172,922 SNPs, we performed an impact analysis using the SnpEff software (http://snpeff.sourceforge.net/). We identified 590 high-impact SNPs (0.1%) leading to exon deletion, frame shift, or loss of a stop codon; 4374 low-impact SNPs (1.1%) leading to synonymous changes in coding regions; and 6335 moderate-impact SNPs leading to non-synonymous changes in coding regions or insertions or deletions of codons. To gain insight into the molecular evolution of the selected SNPs, we investigated the transitions (557,573 total) and transversions (232,492 total), and found a transition/transversion ratio of 2.398. This ratio is similar to a previous study, which reported that the transition/transversion ratio is typically around 2 (Strandberg and Salter 2004).

### Inference of the population structure

**Fig. 2:**
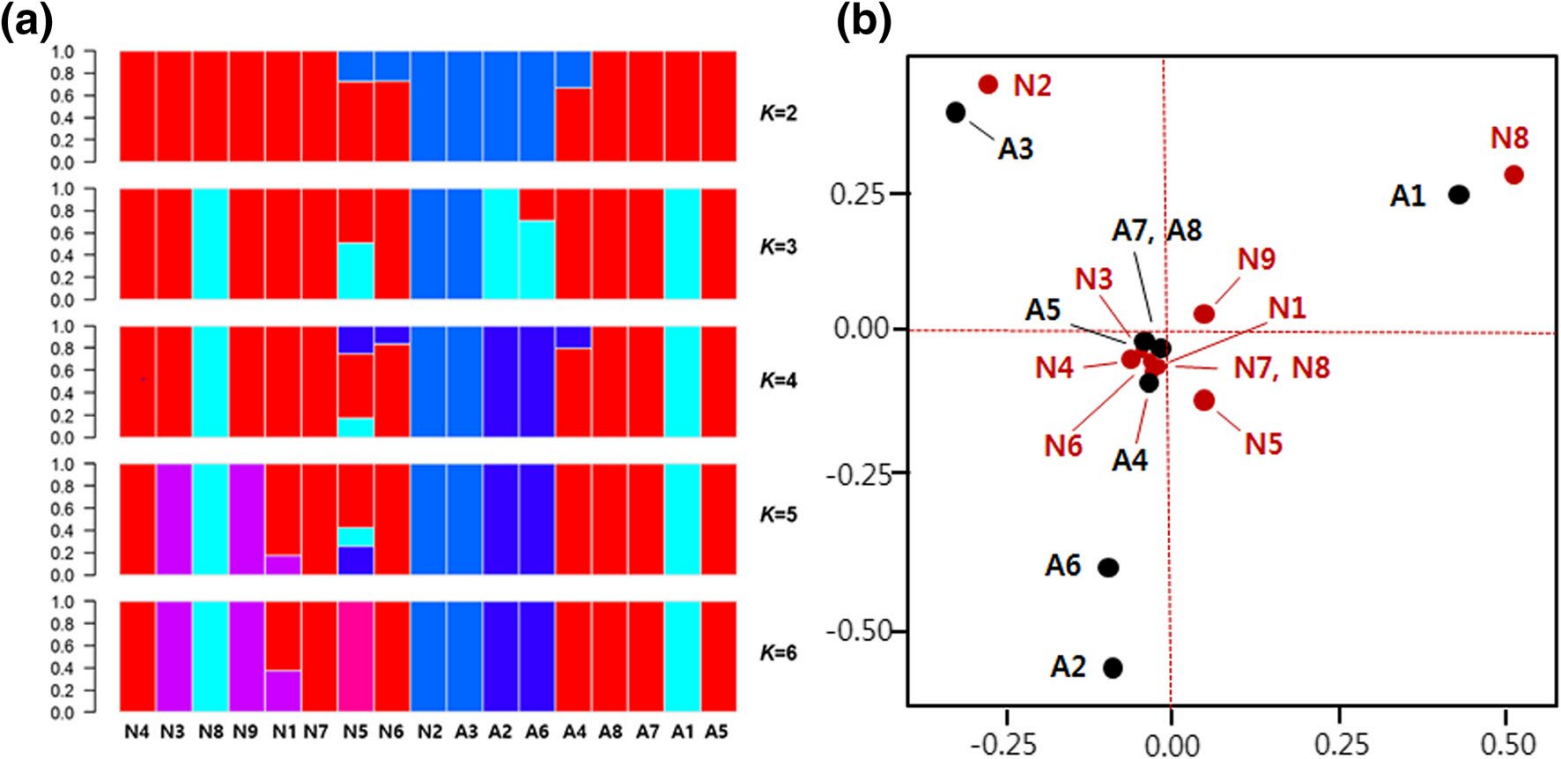
Population structure of the 17 rice accessions on the rice reference genome. **a** A graph of the population structure based on 172,922 single-nucleotide polymorphisms (SNPs) using the FRAPPE program. Each accession is represented by a vertical column. The *K* value represents the cluster number. Clusters of the same color have a similar genetic structure. Red none-anthocyanin genetic structure, blue anthocyanin genetic structure, dark blue flavonoid genetic structure, other colors unknown genetic structure. **b** Results of the principal component analysis. N1–N9 (in red) represent the none-anthocyanin accessions; A1–A8 (in black) represent the high-anthocyanin accessions (black rice, A1–A6; colored rice, A7–A8)

We estimated the population structure using FRAPPE on the 172,922 SNPs. We analyzed ancestry by increasing the number of clusters, *K,* from 2 to 6. At *K* = 2, the non-anthocyanin accessions were not distinctly separated from the high-anthocyanin accessions. At *K* = 4, the A7 and A8 high-anthocyanin accessions (i.e., colored rice) separated from the other high-anthocyanin accessions (i.e., black rice; Fig. 2a). These results suggest that there is no difference in genetic structure between high-anthocyanin and non-anthocyanin accessions despite the presence of a few differences in genetic traits. PCA resulted in a similar conclusion: non-anthocyanin accessions tended to separate from the high-anthocyanin accessions, and the high-anthocyanin accessions showed a high degree of relatedness (Fig. 2b). PCA corrects for population stratification in genome-wide association studies (Price et al. 2006); therefore, we compared the relationship between the two methods. However, we did not obtain significant results for population-specific diversity related to major principal components due to our small sample size.

### Identification of genes based on DNA variation

**Fig. 3:**
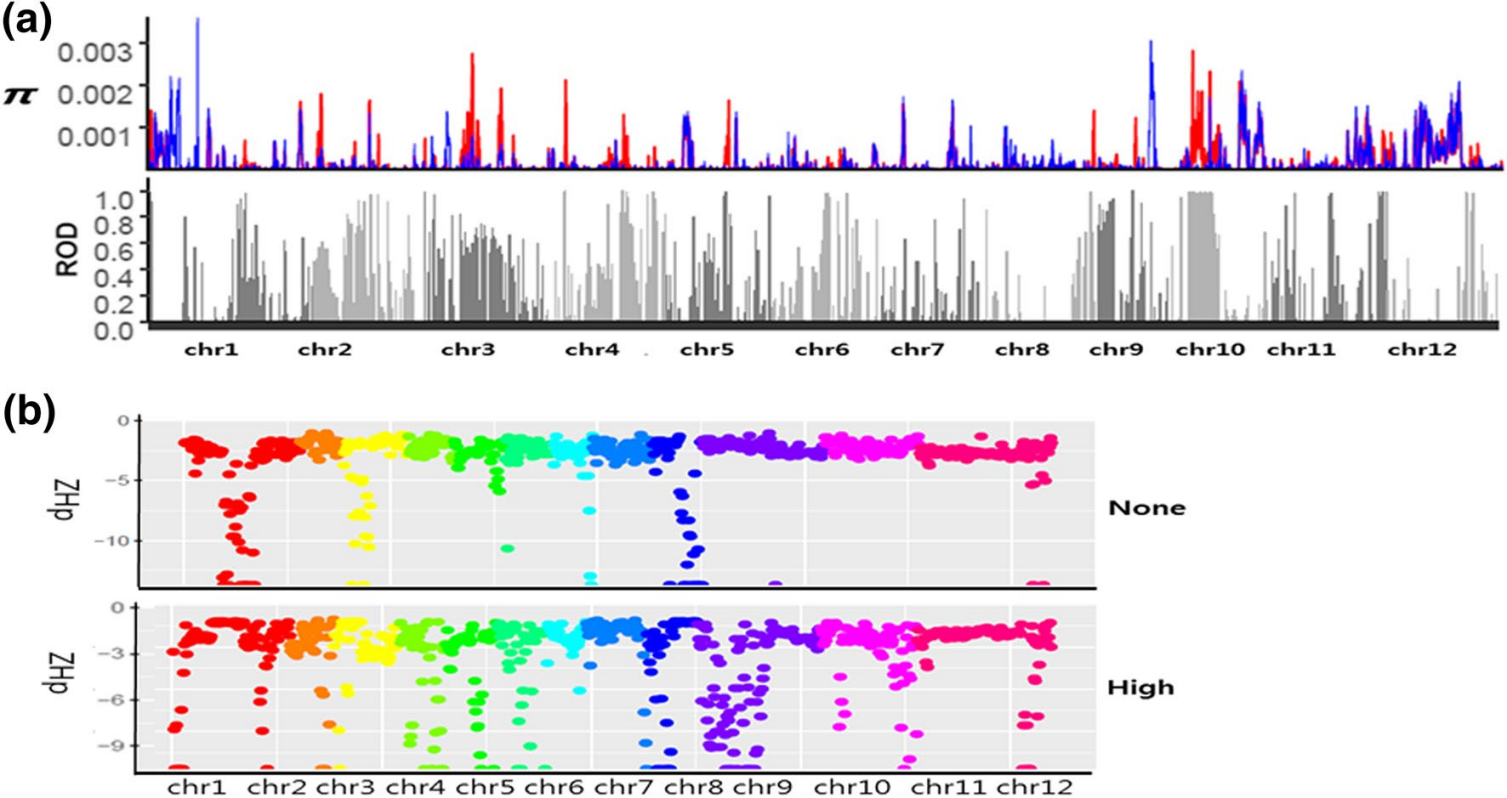
Variation in heterozygosity in the selective sweep regions of rice chromosomes 1–12. **a** The reduction of diversity (ROD) score was calculated in chromosomes 1–12. The blue color indicates the none-anthocyanin group, and the red color indicates the high-anthocyanin group. **b** The Manhattan plots showed intense Z-transformed heterozygosity (ZHp) scores across chromosomes between the none-anthocyanin and high-anthocyanin groups. Each chromosome is distinguished by a different color. The Manhattan plots reveal the low heterozygosity between groups

A selective sweep for anthocyanin production reduces or eliminates variation within encoding genes related to anthocyanin biosynthesis (Olsen et al. 2006). To detect selective sweeps, we used the ROD score for every non-overlapping window of 100 kb along the entire genome with a step size of 20 kb. We identified the genomic regions with positive ROD scores and genetic diversity among both the high-anthocyanin accessions (represented by the summary statistic *π*_high_) and the non-anthocyanin accessions (represented by the summary statistic *π*_none_), and considered those with genetic diversity (*π*) ≤  0.005 to be putative loci related to anthocyanin production. Given that the *π* value measures genetic diversity across accessions and the ROD scores were calculated with a reduced *π* value, genes with high ROD scores are likely to be the causal genes of the common trait. We found high ROD scores on chromosomes 2, 3, and 10, which are expected to be related to the common traits of the anthocyanin accessions (Fig. 3a).

In the high-anthocyanin accession group, we identified 2990 loci with more than one mutation and 141,089 SNPs. In the non-anthocyanin accession group, we identified 2796 loci and 135,966 SNPs. A total of 2685 loci with 172,922 SNPs were identified in common between the non-anthocyanin and the high-anthocyanin groups. The *F*_*ST*_, which is frequently used as a summary of genetic differentiation among groups, depends on the allele frequencies at a given locus and exhibits a variety of peculiar properties related to genetic diversity (Jakobsson et al. 2013). To visualize selective sweeps, we generated Manhattan plots of the *F*_ST_ using the allele counts of the identified SNPs in 100-kb sliding windows that included at least 30 SNPs per window block. The range of heterozygosity was greater among the high-anthocyanin accessions than among the non-anthocyanin accessions. Comparison between the non-anthocyanin and high-anthocyanin accessions showed intense heterozygosity in chromosomes 1, 2, 4, 5, 10, 11, and 12. In the high-anthocyanin group, the plot of the low heterozygosity regions identifies anthocyanin-related traits (Fig. 3b); this plot pattern is similar to the ROD distribution in Fig. 3a. In the non-anthocyanin group, the Manhattan plot identifies traits unique to the non-anthocyanin accessions (Fig. 3b).

**Fig. 4:**
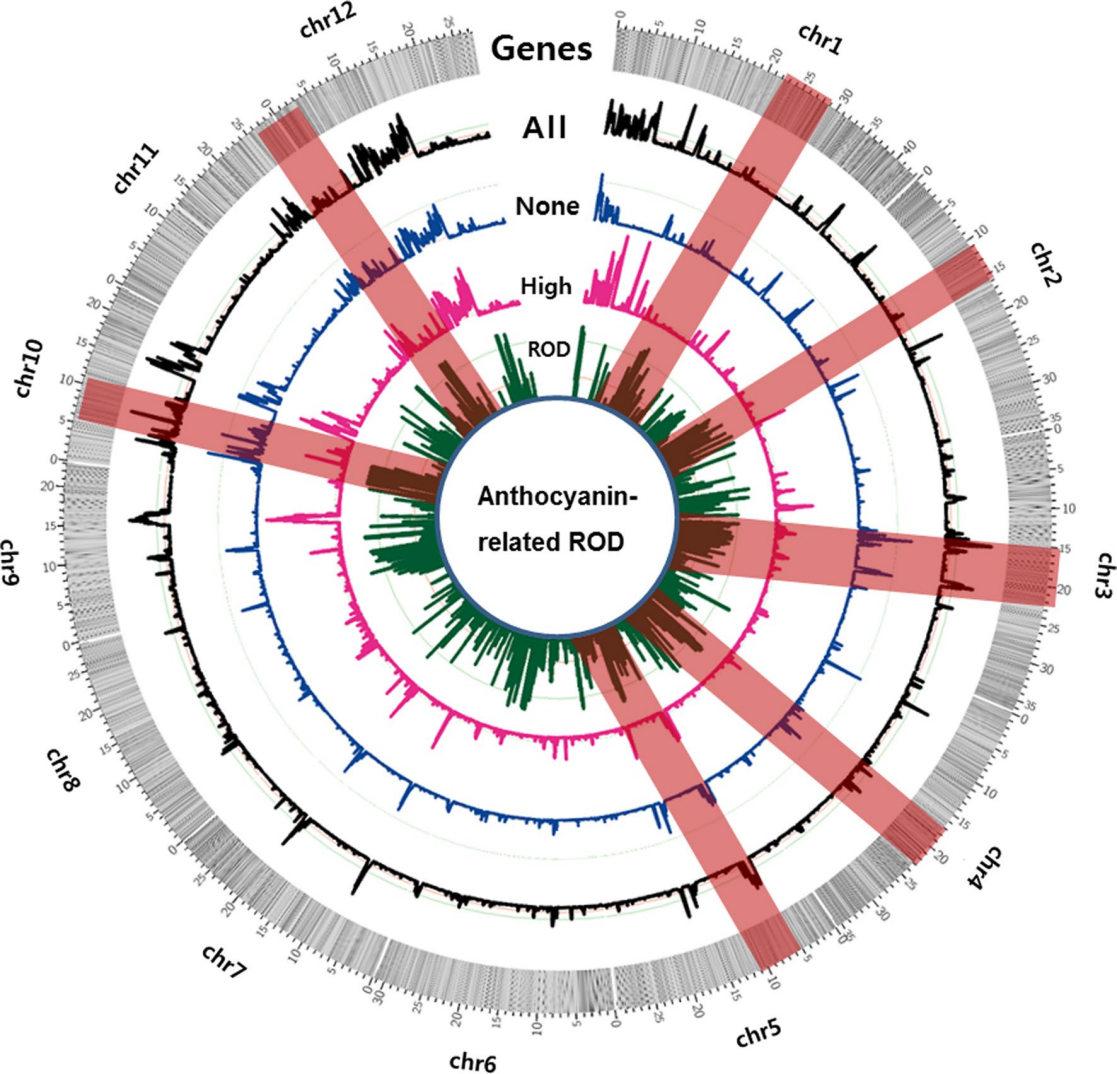
Circos diagram showing the reduction of diversity (ROD) patterns for the non-anthocyanin and high-anthocyanin groups. The Circos diagram was generated using a 5-kb window. The outer ring shows the gene density calculated across all 12 chromosomes. Regions with significant ROD scores are shown with their ROD values. The pink quadrangles show chromosomal regions where there is evidence of intensive selective sweeps. ROD = 1 − (*π*_high_/*π*_none_), where *π*_high_ represents high-anthocyanin rice accessions, and *π*_none_ represents non-anthocyanin rice accessions

To effectively present the regions across all the chromosomes that showed evidence of selective sweeps, we re-plotted the ROD distribution along with the gene density in a Circos diagram using 5-kb windows (Fig. 4). Comparison between the non-anthocyanin and high-anthocyanin accessions showed that intensive selective sweeps occurred in chromosomes 1, 2, 3, 4, 5, 10, and 12. Regions of chromosome 10 showed the greatest differences between the two groups. In addition, the Manhattan plots of heterozygosity and the ROD distributions of selective sweeps showed a similar pattern across all the chromosomes.

To detect putative genes located in the genomic regions that showed evidence of selective sweeps, we screened candidate genes with a ZHp score of − 1.5 or lower. Although we do not detect a strong selective-sweep signal (i.e., threshold ZHp score to − 2.0 or lower) due to a small sample size, we identified 18 genes located in the regions that passed the threshold ZHp score (Table 2). The occurrence of a selective sweep based on small population sizes is likely flawed because of an underestimation of the actual heterozygosity level (Nielsen et al. 2005). Nevertheless, the 18 selected candidate genes can provide useful guidance for the rapid identification of genes in the anthocyanin biosynthesis pathway.

**Table 2:** The 18 predicted genes related to anthocyanin biosynthesis based on single-nucleotide polymorphism (SNP) variations with selective sweeps

RAP-DB gene	Chr.	Start	End	Strand	nSNP^a^	ZHp	Description
*Os04g0175600*	4	5,161,947	5,167,404	+	22	− 1.57	Similar to 0-methyltransferase
*Os04g0175900*	4	5,178,491	5,186,485	+	36	− 1.59	Winged helix repressor domain
*Os04g0176200*	4	5,189,974	5,194,492	–	3	− 1.57	Similar to N-methyltransferase
*Os04g0176300*	4	5,210,111	5,216,557	+	48	− 1.57	Hypothetical protein
*Os04g0176400*	4	5,210,122	5,216,551	–	2	− 1.57	Similar to serine carboxypeptidase 1
*Os05g0338933*	5	15,866,499	15,866,891	+	26	− 1.58	Proton-dependent oligopeptide transport
*Os05g0339000*	5	15,873,838	15,880,419	–	8	− 1.58	VHS domain-containing protein
*Os05g0340000*	5	15,931,964	15,933,657	–	4	− 1.49	Conserved hypothetical protein
*Os10g0162856*	10	4,226,902	4,229,142	+	30	− 1.52	Chalcone and stilbene synthases
*Os10g0174751*	10	5,198,971	5,204,584	+	2	− 1.57	Hypothetical protein
*Os10g0175500*	10	5,236,268	5,237,084	+	43	− 1.59	Hypothetical gene
*Os10g0175700*	10	5,237,690	5,245,182	–	17	− 1.57	Hypothetical protein
*Os10g0175800*	10	5,247,357	5,248,013	+	10	− 1.57	Similar to nodulin protein
*Os10g0188100*	10	6,079,970	6,087,844	–	2	− 1.55	Conserved hypothetical protein
*Os10g0188275*	10	6,103,102	6,109,882	–	3	− 1.56	Hypothetical protein
*Os10g0188400*	10	6,111,498	6,115,269	+	10	− 1.56	Similar to ACI13
*Os10g0188300*	10	6,104,501	6,110,244	+	5	− 1.56	Similar to JHL05D22.13 protein
*Os10g0188900*	10	6,144,589	6,150,509	+	43	− 1.65	Conserved hypothetical protein

^a^Number of SNPs in the gene

### Characteristics of detected genes

We performed 30 non-replicated RNA-seq and 27 microarray experiments on eight high-anthocyanin accessions and two non-anthocyanin accessions (control) at three developmental stages (i.e., 5, 10, and 15 days after heading). We identified the eight high-anthocyanin accessions based on the seed color and leaf color compared with those of the non-anthocyanin accessions. To detect the common genes for comparing RNA-seq and orthologous genes, we first screened 2716 transcripts for expression levels that differed at least twofold between the high-non-anthocyanin accessions during the three time points, and identified 1276 genes that were differentially expressed within at least two of the three time points. Second, to identify conserved orthologous genes associated with anthocyanin biosynthesis, we performed Gene Ontology (GO) enrichment analyses of the 1276 differentially expressed genes (DEGs), which identified 572 orthologous genes belonging to anthocyanin-functional categories. Third, we screened transcriptome genes that were differentially expressed at all time points of high-anthocyanin accessions among 572 orthologous genes. Finally, we identified nine genes that were involved in anthocyanin-related biosynthesis and/or metabolism.

**Fig. 5:**
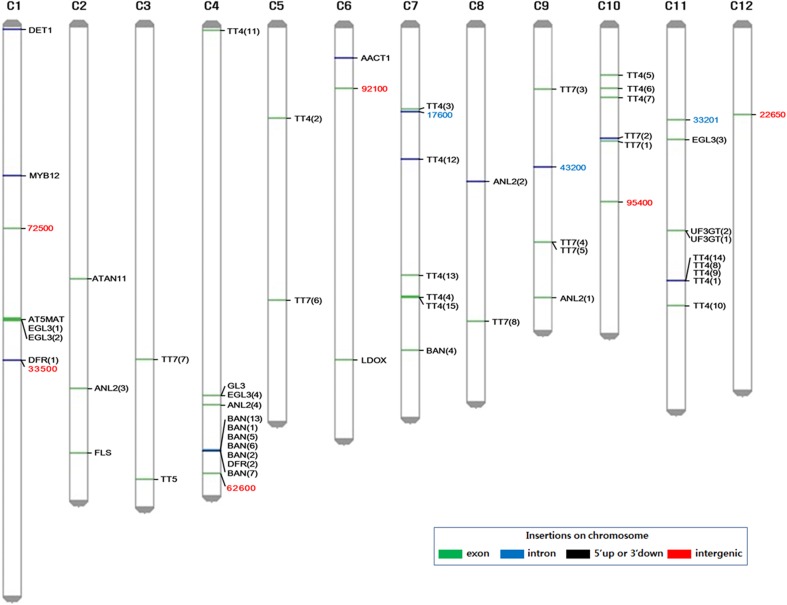
Genetic map of the 12 rice chromosomes showing anthocyanin-related genes, including the nine candidate genes. The black text indicates 16 homologous proteins, which were categorized from 51 pathway genes. The red text indicates six upregulated genes and the blue text indicates three downregulated genes among the nine candidate genes

To predict the functions of the 27 candidate genes (i.e., 18 genes based on selective sweeps and nine genes based on the transcriptome), we checked the gene descriptions and pathways using KEGG and RAP-DB (http://rapdb.dna.affrc.go.jp/). However, the 18 putative genes identified based on the selective sweeps analysis did not show direct evidence for a role in anthocyanin biosynthesis/metabolism (Table 2). Therefore, we checked the characteristics of the nine candidate genes identified by the transcriptome analysis. The mapping results, including the chromosome position, matching trait, and mapping scores, are reported in Table 3. Four of the nine genes showed direct evidence of encoding proteins involved in anthocyanin biosynthesis/metabolism: *Os01t0633500* (Li et al. 2012), *Os01t0372500* (Shih et al. 2008, Lee et al. 2015), *Os04t0662600* (Kim et al. 2008) and *Os06t0192100* (Oikawa et al. 2015). The other five genes did not encode any well-known proteins (Table 4, Fig. 5).

**Table 3:** Mapping matrix of the candidate genes based on the transcriptome and the expression ratio of gene regulation

Transcript	Chr^a^	ML^b^	MM^c^	Gaps	Score	RAP-DB^d^	Regulation	Ratio^e^
*Os01t0372500*	1	1589	0	0	3102	*Os01g0372500*	Up	25.2
*Os01t0633500*	1	1088	0	0	2074	*Os01g0633500*	Up	18.1
*Os04t0662600*	4	2796	0	0	5204	*Os04g0662600*	Up	36.0
*Os06t0192100*	6	1120	0	0	2161	*Os06g0192100*	Up	17.6
*Os07t0217600*	7	2729	0	0	5327	*Os07g0217600*	Down	0.01
*Os09t0343200*	9	6617	0	0	13,120	*Os09g0343200*	Down	0.01
*Os10t0395400*	10	1614	0	0	2926	*Os10g0395400*	Up	27.7
*Os11t0233201*	11	1740	0	0	3354	*Os11g0233201*	Down	0.01
*Os12t0222650*	12	366	0	0	684	*Os12g0222650*	Up	25.3

^a^Chromosome

^b^Matching length

^c^Mismatch

^d^Predicted gene name (http://rapdb.dna.affrc.go.jp/)

^e^Gene expression ratio compared to non-anthocyanin rice

**Table 4:** Characterization of nine genes related to anthocyanin biosynthesis in rice identified by transcriptome experiments

Candidate	Ch^a^	Gene	Protein	Pathway	References
*Os01t0372500*	1	ANS1	Leucoanthocyanidin dioxygenase 1	Flavonoid biosynthesis	Shih et al. (2008), Lee et al. (2015)
*Os01t0633500*	1	Dfr	Similar to dihydro flavonol 4-reductase	Flavonoid biosynthesis	Li et al. (2012)
*Os04t0662600*	4	F3H-1	Flavanone 3-dioxygenase 1	Flavonoid biosynthesis	Kim et al. (2008)
*Os06t0192100*	6	UGT	UDP-glucose flavonoid-3-O-glucosyltransferase	Anthocyanin biosynthesis	Oikawa et al. (2015)
*Os07t0217600*	7	CYP71Z2	CytochromeP450 monooxygenase	Unreviewed	RAP-DB^b^
*Os09t0343200*	9	*Os09g0343200*	Ankyrin repeat containing protein	Unreviewed	RAP-DB
*Os10t0395400*	10	*GSTU34*	Thioredoxin fold domain-containing protein	Unreviewed	RAP-DB
*Os11t0233201*	11	*Os11g0233201*	Hypothetical gene	Unreviewed	RAP-DB
*Os12t0222650*	12	*Os12g0222650*	Hypothetical gene	Unreviewed	RAP-DB

^a^Chromosome

^b^RAP-DB (http://rapdb.dna.affrc.go.jp/)

To reveal the relationship between the nine candidate genes and well-known anthocyanin-related genes, we investigated the anthocyanin-related metabolism of these candidate genes using an enriched-pathway analysis. First, we identified 327 genes with a reported association with anthocyanin biosynthesis in the primary literature (https://www.ncbi.nlm.nih.gov/pubmed/) across all plant species. Second, we screened the 1276 genes identified above from the transcriptome analysis. Third, we used Fisher’s exact test (*p* ≤ 0.05) to determine the most significant network interaction responses using the Pathway Studio^®^ software. Finally, we identified 51 interconnected genes in these network responses. We assumed that the well-characterized homologous genes would more effectively reveal an association with anthocyanin biosynthesis than these 51 hypothetical genes. Therefore, these 51 genes were categorized into 16 well-characterized groups of homologous protein genes (i.e., *AACT1, ANL2, AT5MAT, ATAN11, BAN, DET1, DFR, EGL3, FLS, GL3, LDOX, MYB12, TT4, TT5, TT7,* and *UF3GT*) from the sequenced *A. thaliana* genomes.

### Phylogenetic classification of detected genes

**Fig. 6:**
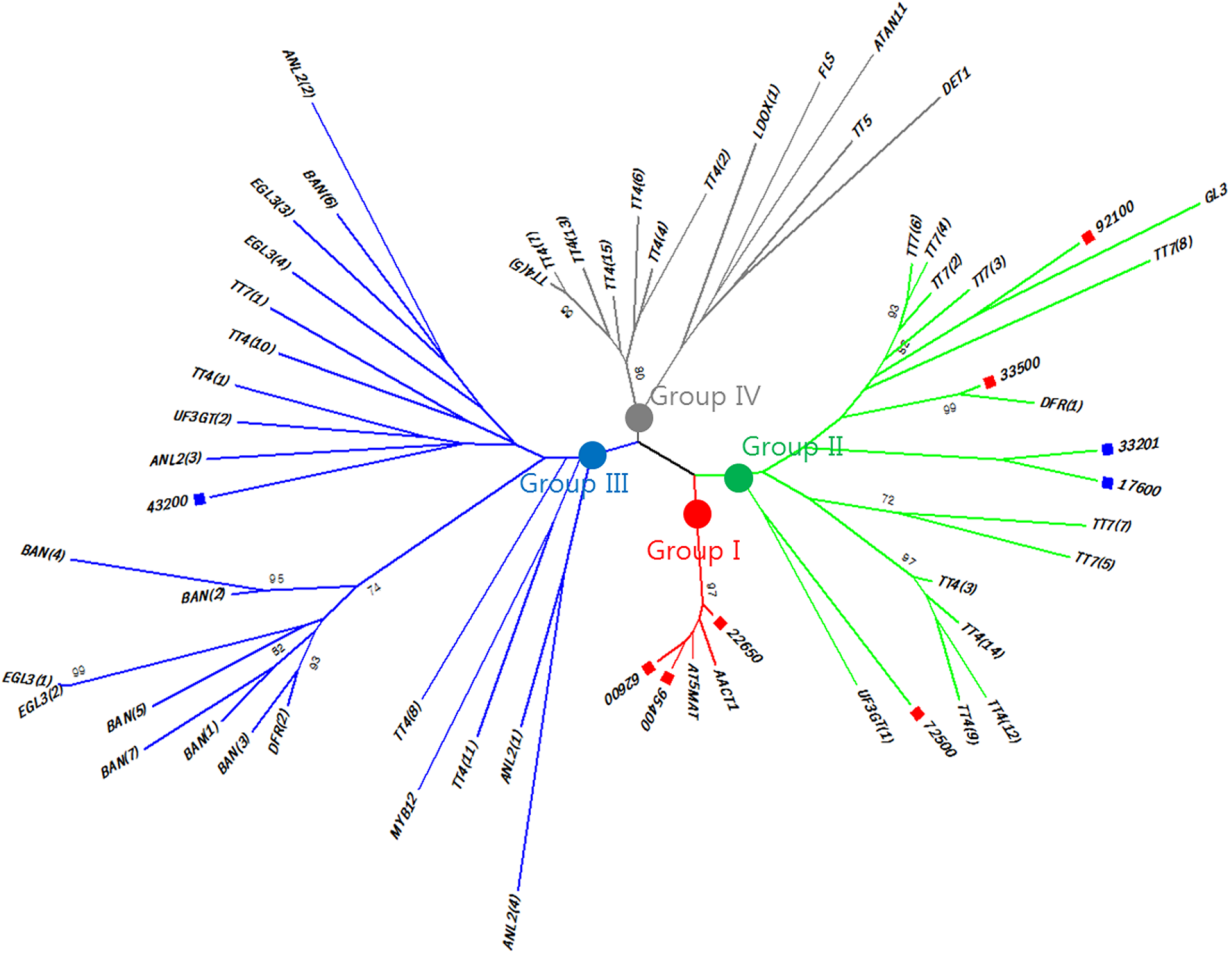
Phylogenetic trees with hierarchical clustering. Phylogenetic trees were generated using our nine candidate genes and 16 homologous proteins categorized from the 51 pathway genes. Of the nine candidate genes, six were upregulated (red squares) and three were downregulated (blue squares). The different line colors represent the four subgroups that were identified

To determine the homologous relationships of our candidate genes, we mapped the nine candidate genes and 16 protein group genes onto the 12 rice chromosomes (Fig. 5), and performed a maximum-likelihood phylogenetic analysis using the MEGA6 software (Fig. 6). A phylogenetic tree with hierarchical clustering was constructed to illustrate the relationships among the 16 protein group genes and the nine candidate genes. We clustered the nine candidate genes into four subgroups (Groups I–IV). Among the nine selected genes, Group I contains only upregulated genes; Group II contains both upregulated and downregulated genes; Group III contains only downregulated genes; and Group IV contains genes that are not related to up- or downregulation (Fig. 6). Group I includes three upregulated genes and two protein genes (i.e., *AT5MAT* and *AACT1*), which positively affect anthocyanin production and accumulation. Group II, III, and IV also include genes that are assumed to affect the gene regulation of anthocyanin production either positively or negatively. However, due to insufficient bootstrapping, we did not find significant hierarchical clustering to identify homologous relationships.

### Comparison of SNP variations and transcriptome

**Fig. 7:**
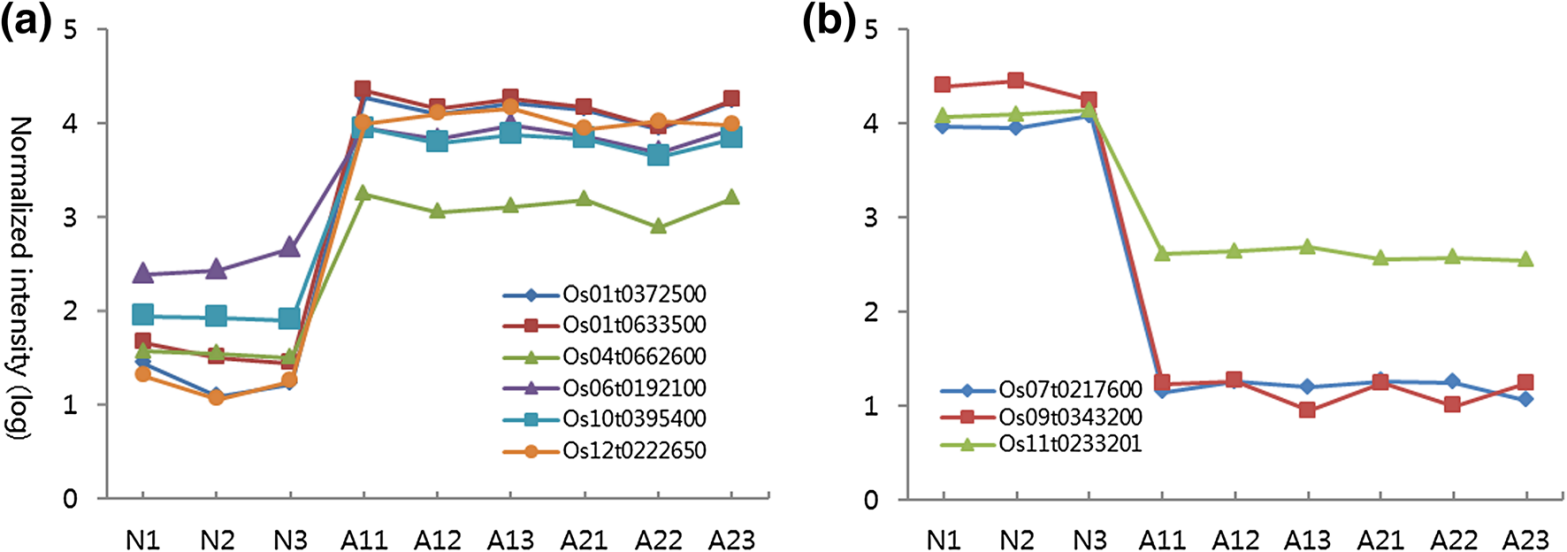
Patterns of gene expression determined from nine selected genes. The x-axis represents N1–N3 (one non-anthocyanin accession at three time points) and A11–A23 (two high-anthocyanin accessions at three time points). **a** The six upregulated genes in the high-anthocyanin group are significantly different from the non-anthocyanin group at all three time points. **b** The three downregulated genes are shown at three time points

Among the nine genes, *Os01t0633500, Os06t0192100, Os01t0372500, Os12t0222650*, *Os04t0662600*, and *Os10t0395400* were significantly upregulated (Fig. 7a), and *Os07t0217600, Os09t0343200*, and *Os11t0233201* were significantly downregulated (Fig. 7b) in the high-anthocyanin accessions. We hypothesize that the amino acid changes caused phenotypic differences in anthocyanin biosynthesis by affecting post-translational processes such as DNA methylation and histone modification events, protein–protein interactions, and metabolism turnover. Although we did not determine the relationship between the SNPs and the transcription levels, it is likely that both genetic variation and gene expression play important roles in causing the phenotypic differences between high-anthocyanin cultivars and non-anthocyanin cultivars. None of the 18 putative genes identified by selective sweep were significantly up- or downregulated on the nine transcriptome genes.

### Verification using semi-quantitative RT-PCR

**Fig. 8:**
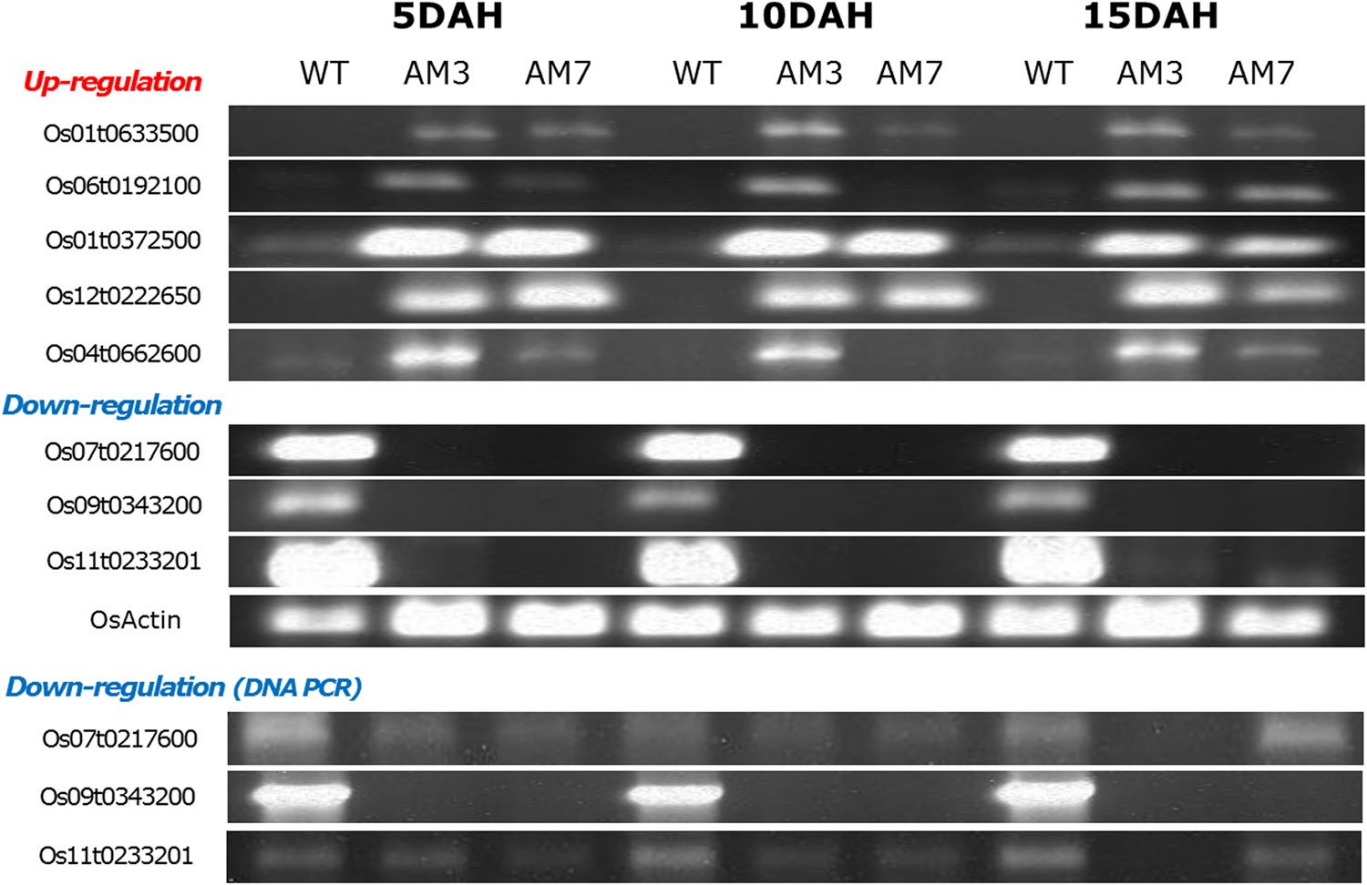
Semi-quantitative reverse-transcriptase polymerase chain reaction **(**RT-PCR) analysis for verification of candidate genes using the same samples used in the rice microarray experiments. In the downregulated genes, DNA-PCR was performed to determine whether primers were not working due to sequence mismatches. *DAH* day after heading, *WT* non-anthocyanin accession (leaves, seed), *AM3* high-anthocyanin accession (seed), *AM7* high-anthocyanin accession (leaves)

The nine candidate genes from RNA-seq analysis were verified by the semi-quantitative RT-PCR using the same samples used in the rice microarray experiments. Five of the genes that were upregulated (*Os01t0633500, Os06t0192100, Os01t0372500, Os12t0222650*, and *Os04t0662600*) most likely either play a regulatory role in anthocyanin production or are related to signaling during anthocyanin biosynthesis. However, the *Os10t0395400* gene did not show a significant expression pattern in the microarray experiments. The three downregulated genes (*Os07t0217600, Os09t0343200*, and *Os11t0233201*) may inhibit anthocyanin biosynthesis. To confirm the possibility that the primers were not working due to sequence mismatches, however, downregulated genes were also evaluated by DNA-PCR. Although the *Os11t0233201* gene exhibited an unclear pattern, overall the expression patterns derived from DNA-PCR were similar to those from RNA-PCR (Fig. 8). We previously assumed that SNP variation and up- or downregulated genes were related to major biological changes induced by the anthocyanin biosynthesis pathway. In this study, however, SNP variation was not significantly correlated with the RNA-seq or microarray expression data; only the RNA-seq and microarray expressions exhibited significant correlations.

In our study, three of the nine candidate genes showed direct evidence for a role in anthocyanin biosynthesis. In particular, the *Os06t0192100* gene (i.e., UDP-glucose flavonoid-3-O-glucosyltransferase) was assumed to be closely related to the *Os04g0557500* gene, which Oikawa et al. previously reported (Oikawa et al. 2015). In previous studies, pigmentation was determined by the functional activities of flavonoid biosynthesis genes (Maeda et al. 2014), population structure associated with genetic diversity (Choudhury et al. 2014), and selective sweeps (Ding et al. 2011). However, we did not obtain significant results for population-specific diversity related to anthocyanin phenotype analysis due to our small sample size. Although the identified genes based on the SNP variation require additional validation for population structure, our study demonstrates the potential of our screening method combining SNP variation and transcriptome data to identify putative genes that play a role either in anthocyanin production or in the control of anthocyanin levels. Further investigation to determine the phylogenetic evolution and gene pathways will be important to expand our understanding of the evolutionary biology of anthocyanin production in rice breeding.

## Electronic supplementary material

Below is the link to the electronic supplementary material.

Supplementary material 1 (DOCX 172 kb)
