# Supplementary figures and images for: Whole-genome resequencing and transcriptomic analysis of genes regulating anthocyanin biosynthesis in black rice plants
Source: 3 Biotech. 2018 Feb 7;8(2):115. doi: 10.1007/s13205-018-1140-3 (PMC5801106; doi:10.1007/s13205-018-1140-3)

**Supplementary data**

**
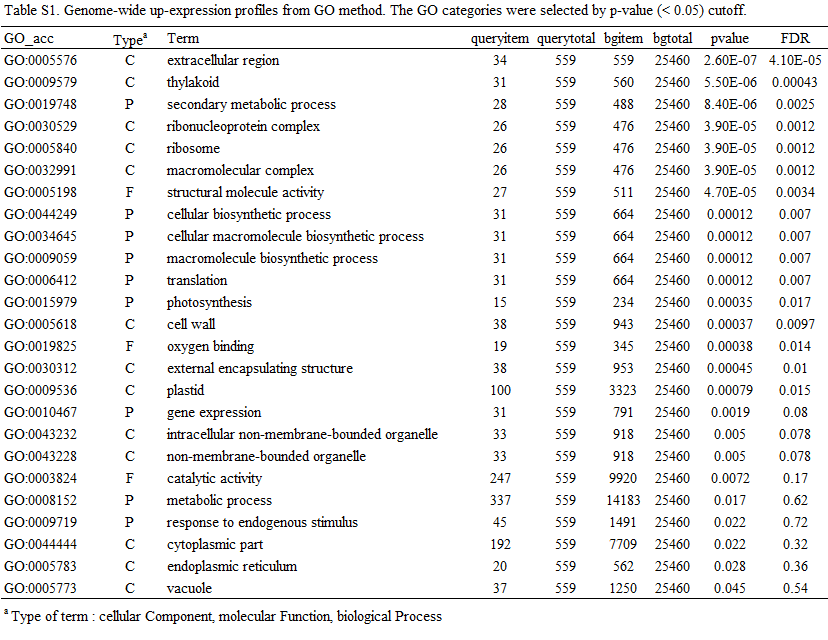
**

**
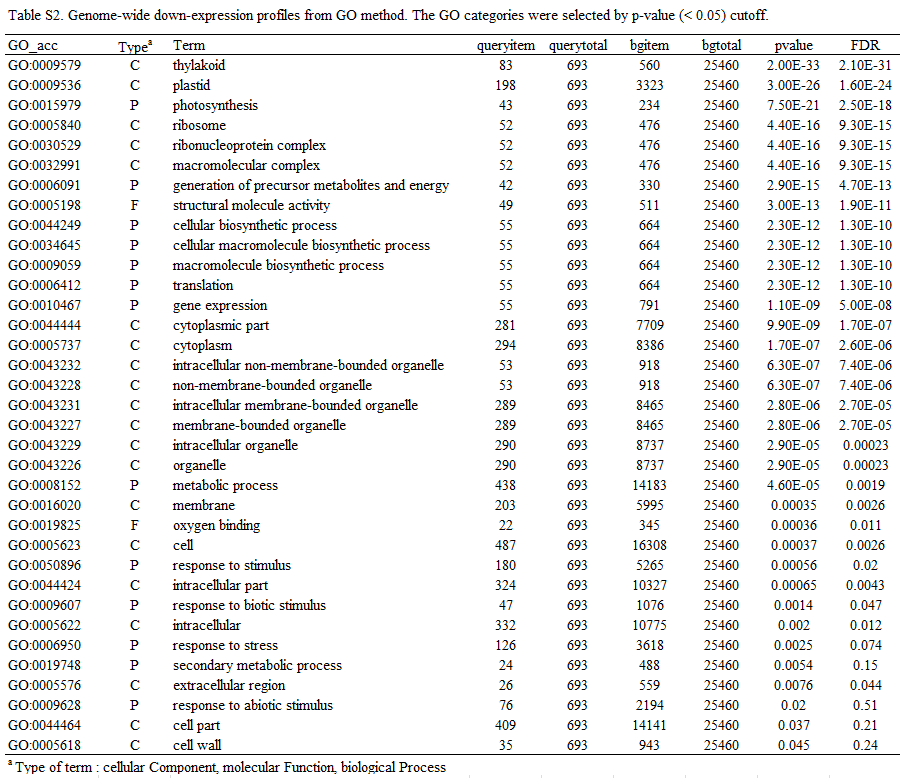
**

Supplement: Supplementary file 1 — Supplementary material 1 (DOCX 172 kb) [file 13205_2018_1140_MOESM1_ESM.docx]
